# Prognostic Value of a CT Radiomics-Based Nomogram for the Overall Survival of Patients with Nonmetastatic BCLC Stage C Hepatocellular Carcinoma after Stereotactic Body Radiotherapy

**DOI:** 10.1155/2023/1554599

**Published:** 2023-01-03

**Authors:** Lihong Wang, Danfang Yan, Liang Shen, Yalin Xie, Senxiang Yan

**Affiliations:** ^1^Department of Radiation Oncology, The First Affiliated Hospital, Zhejiang University School of Medicine, Hangzhou, China; ^2^Hepatopancreatobiliary Surgery Department, The First Affiliated Hospital, Zhejiang University School of Medicine, Hangzhou, China; ^3^Department of Radiotherapy, Hangzhou Cancer Hospital, Hangzhou, China

## Abstract

**Purpose:**

This study aimed to investigatie the feasibility of pretherapeutic CT radiomics-based nomograms to predict the overall survival (OS) of patients with nondistant metastatic Barcelona Clinic Liver Cancer stage C (BCLC-C) hepatocellular carcinoma (HCC) undergoing stereotactic body radiotherapy (SBRT).

**Methods:**

A retrospective review of 137 patients with nondistant metastatic BCLC-C HCC who underwent SBRT was made. Radiomics features distilled from pretherapeutic CT images were selected by the method of LASSO regression for radiomics signature construction. Then, the clinical model was constructed based on clinical characteristics. A radiomics nomogram was constructed using the radiomics score (Rad-score) and clinical characteristics to predict post-SBRT OS in BCLC-C HCC patients. An analysis of discriminatory ability and calibration was performed to confirm the efficacy of the radiomics nomogram.

**Results:**

In order to construct the radiomic signature, seven significant features were selected. Patients were divided into low-risk (Rad-score < −0.03) and high-risk (Rad-score ≥ −0.03) groups based on the best Rad-score cutoff value. There were statistically significant differences in OS both in the training set (*p* < 0.0001) and the validation set (*p*=0.03) after stratification. The *C*-indexes of the radiomics nomogram were 0.77 (95% CI: 0.72–0.82) in the training set and 0.71 (95% CI: 0.61–0.81) in the validation set, which outperformed the clinical model and radiomics signature. An AUC of 0.76, 0.79, and 0.84 was reached for 6-, 12-, and 18-month survival predictions, respectively.

**Conclusions:**

The predictive nomogram that combines radiomic features with clinical characteristics has great prospects for application in the prediction of post-SBRT OS in nondistant metastatic BCLC-C HCC patients.

## 1. Introduction

Hepatocellular carcinoma (HCC) is an important challenge for oncologists. Despite introducing a screening program for at-risk populations, the diagnosis of HCC is usually defined at an advanced stage and is not suitable for operative treatment [1]. The Barcelona Clinic Liver Cancer staging system (BCLC) makes treatment recommendations for HCC according to cancer-related symptoms, tumor burden, and liver reserve function [2]. Stage C of BCLC (BCLC-C) represents the advanced stage that is defined by macrovascular invasion and/or extrahepatic extension, mild-to-moderate impairment of liver function, and performance status (PS) 1–2 [2]. Patients in the advanced stage are generally treated with systemic therapy, such as immunotherapy with anti-VEGF antibodies and targeted therapy; however, response rates are modest [3–5]. There is still a paucity of clinical studies required to reach a consensus on the choice between systemic and locoregional treatment at this stage [6]. Appropriate treatment may vary depending on whether there is an existing macrovascular invasion or metastatic lesions. Individualized treatment of patients with BCLC-C HCC remains an important clinical issue.

As one of the locoregional treatment options for HCC, stereotactic body radiation therapy (SBRT) is listed in the National Comprehensive Cancer Network (NCCN) Guidelines for inoperable HCC according to recent advances [7]. A prospective study has shown that transarterial chemoembolization (TACE) plus SBRT improved overall survival (OS) compared to systemic therapy with sorafenib in HCC patients in advanced stages [8]. Another study reported that in HCC patients with regional lymph node or distant metastases, combined SBRT of the intrahepatic HCC lesions demonstrated superior survival OS compared to those treated systemically only [9]. In addition, the combination of SBRT and immunotherapy achieved favorable control (CR rate: 50%) in patients with BCLC-C HCC, even though only a nonablative dose of radiation was delivered because of the extensive tumor burden [10]. SBRT sequenced with systemic or TACE was conditionally recommended for patients with BCLC-C HCC by some clinical evidence-based treatment guidelines [11, 12]. However, there are significant variations in treatment outcomes among BCLC-C HCC patients. So, a reliable nomogram for these patients is vital for providing accurate outcome estimations. Radiomics technology can provide radiomic features, which are quantitative image features, from common medical images [13]. Radiomic features could provide information on the tumor's phenotype and microenvironment, complementary to other clinical data sources [14]. Many radiomic research studies try to predict the efficacy of specific treatment modalities in HCC [15–17]; however, there is no radiomics-based study on the prediction of post-SBRT OS in patients with HCC.

This research constructed a nomogram including the Rad-score and clinical risk factors to predict the post-SBRT OS of nondistant metastatic BCLC-C HCC patients.

## 2. Materials and Methods

### 2.1. Patient Selection

All the patients (*n* = 137) in this study were enrolled at the First Affiliated Hospital of Zhejiang University School from December 2016 to September 2020. The eligibility criteria were as follows: (1) a clinical or pathological diagnosis of HCC based on the widely acknowledged diagnostic criteria [18]; (2) BCLC-C HCC without distant metastasis; (3) tumor confirmation via contrast-enhanced CT within one month before SBRT administration; (4) no history of radiotherapy. The patient selection flowchart is revealed (Supplementary Figure 1).

### 2.2. SBRT

The following indications for SBRT were established by a multidisciplinary tumor panel: (1) combination with TACE and/or systemic therapy; (2) ineligibility or progression after TACE or systemic therapy; and (3) an alternative to systemic therapy. The radiation targets were intrahepatic and macrovascular tumors. All tumors were included in the radiation field if possible. Adjustments were made to the radiation field in some patients depending on the tumor size, normal liver volume, and the CTP score. A radiation dosage of 25–50 Gy was given in 5–8 fx (BED 37.5–100 Gy, with *α*/*β* of 10). We prescribed the radiation dose based on the Child-Turcotte-Pugh (CTP) grade, the dose of 700 cm^3^ for the uninvolved liver, and the distance from the luminal organs.

### 2.3. Image Acquisition

All patients received contrast-enhanced CT imaging using a LightSpeed RT 16 scanner (Phillips Brilliance) before SBRT. The technical parameters of scanning were shown as follows: tube voltage, 120 kVp; rack rotation time, 0.6 s; FOV, 450–550 mm; matrix, 512 *∗* 512; slice thickness, 0.25 cm.

### 2.4. Segmentation of ROI and Radiomic Feature Extraction

Arterial-phase enhanced CT images were imported from PACS into the software program “3D Slicer” (version 4.11) for intrahepatic tumor contouring, which is also referred to as the region of interest (ROI) segmentation. The ROI segmentation was executed by one radiation therapist with ten years of clinical experience and revealed in Supplementary Figure 2.

Three-dimensional (3D) radiomics features of the segmented ROI were obtained by the “Pyradiomics” package of the Python program. To assess the radiomic features' reliability, two radiologists independently delineated the ROIs of 30 randomly chosen patients. ICC was calculated from the extracted features using the “irr” package in the *R* software program to show the reproducibility of the radiomics feature. Features with ICC values below 0.70 were excluded by subsequent feature selection.

### 2.5. Feature Selection and Radiomics Signature Building

The study participants were randomly assigned to the training set (70%) and the validation set (30%). Following the methods of Hong et al. [19], the LASSO Cox regression model was performed to get the prognostic radiomics features from the training datasets, which was a commonly used method for selecting features from high-dimensional radiomics data. The Rad-score of each participant was calculated by weighting the selected features based on their respective coefficients [19].

### 2.6. Validation of the Radiomics Signature

The association between the Rad-score and post-SBRT survival was evaluated via the Kaplan–Meier survival analysis. Patients were assigned into high- and low-risk groups based on the Rad-score cutoff point [19]. The best cutoff point was calculated on the basis of training datasets applying the “survminer” package and tested on validation datasets. Value assessment of radiomics-based risk stratification was conducted by the log-rank test.

Furthermore, ROC curves were generated, and AUCs were calculated to assess the efficacy of radiomic signature survival prediction at 6-, 12-, and 18-month time points. The calculation of the *C*-index was made to assess discrimination power.

### 2.7. Clinical Model Building and Validation

The clinical model was built by the Cox regression analysis [19]. In the training set, sex, age, ECOG, the N stage, BED, MVI, the CTP class, hepatitis, pre-SBRT AFP, the largest tumor size, and the number of tumors were evaluated based on univariate Cox regression analysis. Only the clinical elements with *p* < 0.05 were adopted for further multivariate Cox regression analysis. To evaluate the efficacy of clinical model prediction, the same method was applied to the radiomic signature to generate ROC curves and calculate AUCs and *C*-index [19].

### 2.8. Development and Validation of a Radiomics Nomogram

Following the methods of Hong et al. [19], the Cox regression analyses were conducted to confirm the prognostic characteristics related to post-SBRT OS. The development of the nomogram was made according to the multivariate COX regression model of the Rad-score and clinical characteristics, to provide visualizing evaluation for the post-SBRT survival.

The efficacy of the nomogram prediction for post-SBRT OS was evaluated by the ROC curve and AUCs. The calculation of the *C*-index was made to assess discrimination power. Calibration curves were generated for evaluating the agreement between the actual and estimated post-SBRT OS. A decision curve analysis was also performed to confirm the practicability of the developed nomogram by revealing the net benefits for each threshold probability.

### 2.9. Statistical Analysis

The OS was the time interval from SBRT to death or last follow-up. The comparison of categorical and numerical variables was made with the *x*^2^ test or the Fisher exact test. Survival curves were generated by the Kaplan–Meier analysis and compared by the log-rank test. This study used the R software program (version 4.1.2) for statistical analyses. For all tests, statical significance was defined as *p* < 0.05 [19].

## 3. Results

### 3.1. Characteristics of the Study Participants

This retrospective analysis included 137 patients (17 females and 120 males) with HCC. The participants were assigned to a training set (95) or validation (42) set. The median age of training and validation set participants was between 54 and 58. The median OS of the two sets was 10 and 9 months, respectively. Clinical features were well balanced between the two sets, with *p* values ranging between 0.08 and 1.00 (Table 1).

### 3.2. Feature Selection and Radiomics Signature Building

**Figure 1:**
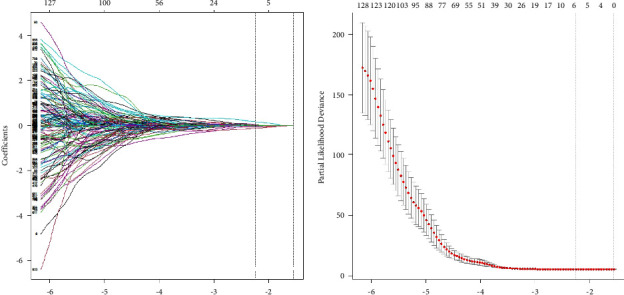
Feature selection. (a) Parameter tuning of the lambda in the LASSO regression. (b) The drawing of the dotted vertical line was made at the value of lambda −2.14 with the minimum partial likelihood of deviance.

**Figure 2:**
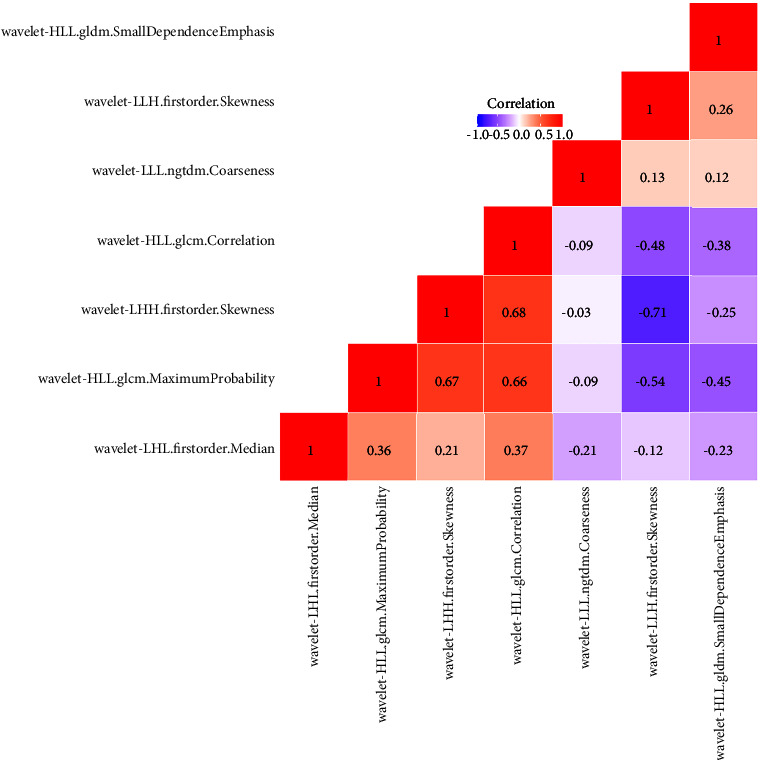
The correlation heat map of radiomic features.

851 features were distilled from each ROI. The reproducibility of radiomic features was assessed by ICC values. 537 radiomics features with ICC of >0.75 were included in the further selection. Based on the LASSO model for OS, seven radiomic features were selected (Figure 1). Spearman's rank correlation coefficient was computed to evaluate correlations between the selected radiomic features. There were no significant correlations with values less than 0.75 (Figure 2). The Rad-scores of each patient were calculated by weighting selected features in the following formula:

(1) $$\begin{array}{c} {Rad-score=0.0303*wavelet-LLH}_{firstorde\;r.Skewness}\\ {+0.0285*wavelet-LHL}_{firstorde\;r.Medi\;an}\\ {-0.0806*wavelet-LHH}_{firstorde\;r.Skewness}\\ {-0.0236*wavelet-HLL}_{glcm.Correlation}\\ {-0.0481*wavelet-HLL}_{glcm.MaximumProbability}\\ {+0.1635*wavelet-HLL}_{gldm.SmallDepende\;nceEmphasis}\\ {-0.1299*wavelet-LLL}_{ngtdm.Coarseness}\text{.} \end{array}$$

### 3.3. Validation of the Radiomic Signature

**Figure 3:**
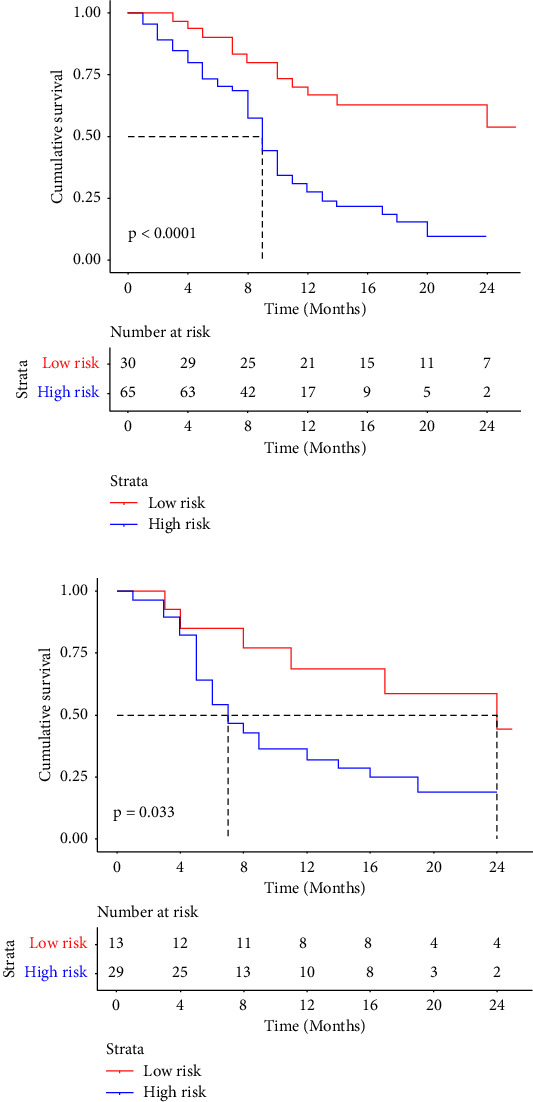
Kaplan–Meier curves demonstrated that Rad-score could efficiently distinguish low-risk and high-risk patients. (a) Training set. (b) Validation set.

All participants were stratified into two groups on the basis of the best cutoff value: patients with Rad-scores of ≥−0.03 were distributed to the high-risk group, while others with scores of <−0.03 were distributed to the low-risk group. Both in the training set (*p* < 0.0001) and the validation set (*p*=0.033), the difference in survival between the two subgroups was statistically significant, as shown in Figure 3.

**Figure 4:**
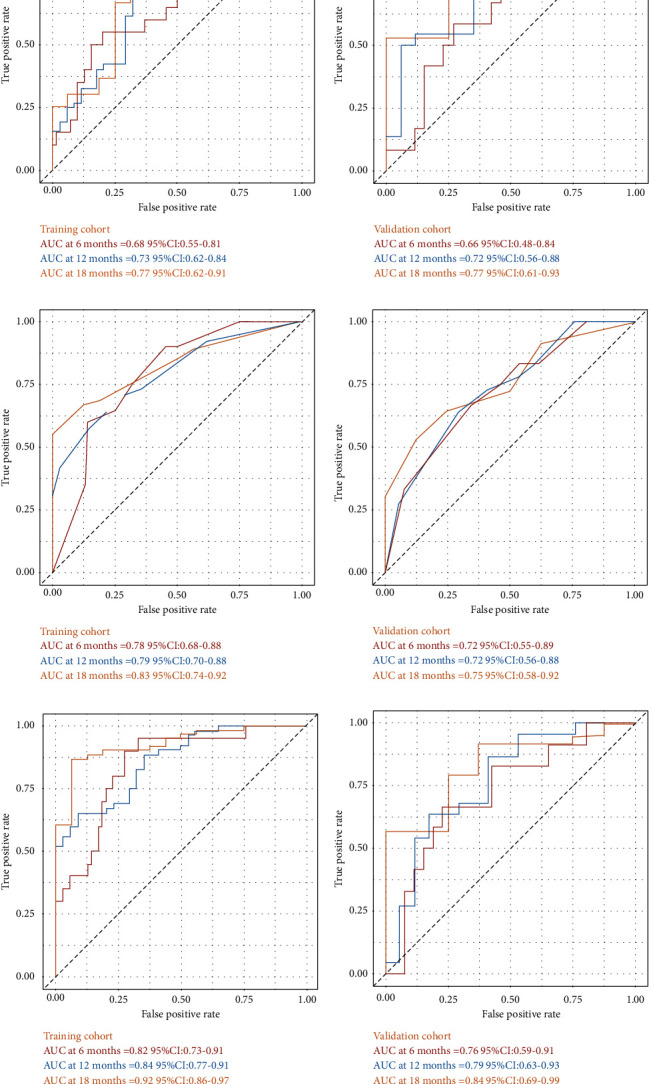
The ROC curves of the two subgroups for 6-, 12-, and 18-month survival in all models. (a) Radiomic signature. (b) Clinical model. (c) Nomogram.

The *C*-indexes of the radiomics signature in the training and validation sets were 0.67 (95% CI: 0.59–0.74) and 0.65 (95% CI: 0.54–0.77). The ROC curves and AUC outcomes of the two sets for 6-, 12-, and 18-month post-SBRT OS prediction are revealed in Figure 4(a).

### 3.4. Clinical Model Building and Validation

Clinical characteristics with a *p* < 0.05 according to the univariate Cox analysis (Table 2) were adopted by the multivariate analysis. The largest tumor size, CTP class, and BED were confirmed to be prognostic factors for post-SBRT OS based on the proportional hazard model.

The *C*-indexes of the clinical model were 0.72 (95% CI: 0.66–0.78) and 0.67 (95% CI: 0.57–0.77) in the training and validation sets, respectively. The ROC curves and AUC outcomes of the two subgroups for 6-, 12-, and 18-month survival after SBRT are illustrated in Figure 4(b).

### 3.5. Construction and Validation of the Radiomics Nomogram

**Figure 5:**
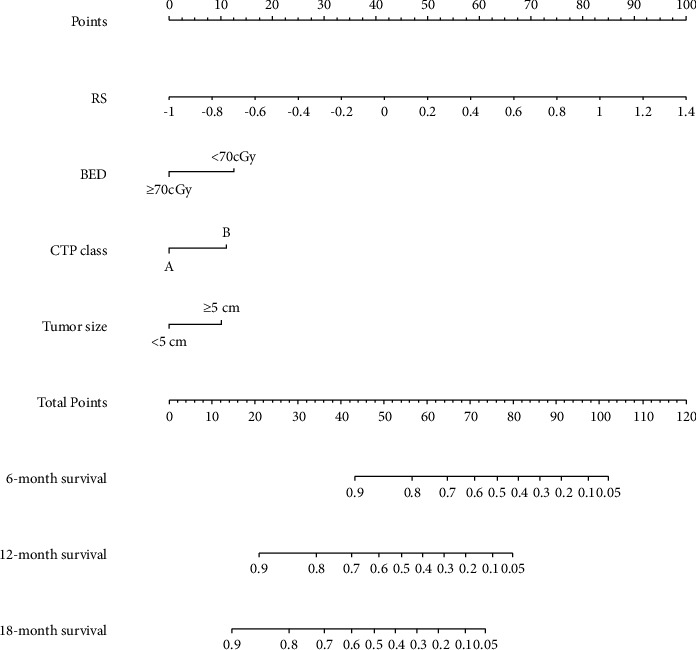
Nomogram for predicting the 6-, 12-, and 18-month overall survival rates.

**Figure 6:**
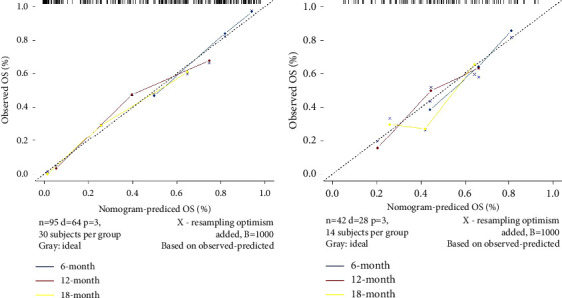
Calibration curves presented a high level of agreement between the predicted outcomes of the nomogram and the actual 6-, 12-, and 18-month post-SBRT survival rates. (a) Training set. (b) Validation set.

**Figure 7:**
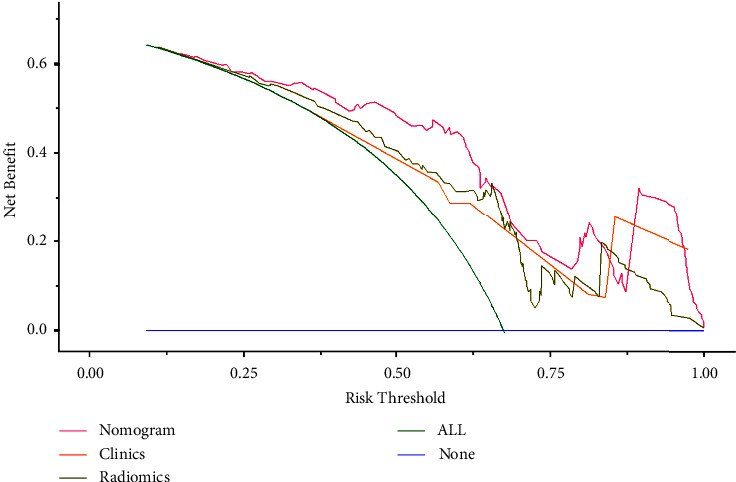
The decision curve of each model.

Multivariate Cox regression analyses (Table 2) revealed that the largest tumor size, CTP class, BED, and Rad-score were predictors of post-SBRT OS for BCLC-C patients. Based on this, a nomogram including the clinical characteristics and the Rad-score was developed (Figure 5) to predict the overall survival rate after SBRT for the nondistant metastatic BCLC-C HCC patients. The nomogram demonstrated that the radiomics signature had the largest impact on OS. The largest tumor size, CTP class, and BED were shown to have a moderate impact on OS. The *C*-indexes of the radiomics nomogram were 0.77 (95% CI: 0.72–0.82) in the training set and 0.71 (95% CI: 0.61–0.81) in the validation set. The ROC and AUC outcomes of the two sets for 6-, 12-, and 18-month post-SBRT prediction are shown in Figure 4(c). The calibration curve presented a high level of agreement between the predicted outcomes of the combined model and the actual 6-, 12-, and 18-month post-SBRT overall survival rate (Figure 6). As the decision curve analysis of the radiomics nomogram revealed (Figure 7), the radiomics nomogram acquired the greatest net benefit compared to “treat-all” or “treat-none” schemes for all threshold probabilities. We also compared the performance of all models with the nomogram (Table 3).

## 4. Discussion

BCLC-C HCC is a group of cancers with huge heterogeneity. To date, there is still a paucity of clinical studies needed to reach a consensus on the choice between systemic and locoregional treatment at this stage. It is important to investigate the individual basis of prognostic factors and treatment options for these groups. More and more published studies have demonstrated the benefit of integrating SBRT with other treatment modalities [8–10]. We established and validated a nomogram to show these patients' 6-, 12-, and 18-month post-SBRT survival probabilities. Pretreatment information, including the Rad-score and clinical prognostic elements, was integrated into this monogram. Thus, a personalized estimation can be made for the potential benefit of SBRT before the clinician's decision.

Both liver function and intrahepatic tumor burden are clinical prognosticators in our model, which are reflected by the CTP class and largest tumor size, respectively. These results are in agreement with previously reported clinical models [20, 21]. In contrast to the findings reported in previous articles [22, 23], MVI (*p*=0.16) was not a clinical prognosticator in our study because all the patients with macrovascular tumors underwent SBRT, which can result in improved local control and a higher OS compared to historical controls [24]. Consistent with the conclusion of former research [25], we found that SBRT with a BED of ≥70 Gy provides better oncological outcomes in local advanced HCC. In comparison, the increase in BED per 1 Gy lost significance in the univariate analysis of OS. These findings suggest that a dose higher than the threshold BED of 70 Gy is required for better treatment outcomes. The nomogram (Figure 5) illustrated the moderate effect of tumor size, BED, and CTP class on OS.

In this study, the Rad-score established by pre-SBRT enhanced CT images had the largest contribution to the prediction of the OS (HR = 10.6, *p* < 0.001). There are seven radiomic features in the radiomic signature, four of which are texture-based, offering tumor heterogeneity information for prediction. High textural heterogeneity can negatively influence survival [14]. Patients were divided into high- and low-risk groups based on the best cutoff point of the Rad-score OS, which was significantly different between the two risk stratification groups in both the training and validation sets.

Many complementary researchers have tried to construct a radiomics-based model to forecast the efficacy of HCC with other treatment modalities. Chen et al. proved that radiomic features of MRI images could forecast the outcomes in patients with immunotherapy-treated HCC [26]. Shan et al. set up a radiomics-based model capable of efficiently forecasting the early recurrence of HCC after curative tumor treatment [15]. Li et al. used texture analysis to perform stratifications of HCC patients to determine the appropriateness of liver resection versus TACE [27]. The results of these studies showed that radiomic features—noninvasive, quantitative, and low-cost parameters—were invaluable for forecasting the outcome of HCC.

The Ras-score and the clinical model developed by this study had similar predictive capacities in the validation set (*C*-index value of 0.66 and 0.67), and they were found to complement each other in forecasting OS. With the integration of the Rad-score and clinical elements, the nomogram reached a *C*-index of 0.71 in the validation set. In the training set, the nomogram displayed significantly better performance compared to both the radiomics signature (*p* < 0.01) and the clinical model (*p* < 0.05), and there was a tendency for the integrated model to have a better effect than the single model in the validation set (Table 3). The nomogram had the highest AUCs for 6-, 12-, and 18-month OS predictions among these models (Figure 4). The AUC of our nomogram for 12-month OS was 0.79, which was better than the reported clinical risk factor-based nomogram for predicting 12-month OS (AUC 0.74) [28]. It is encouraging that our research is the first to explore the application of radiomics for the prediction of post-SBRT OS in patients with nondistant metastatic BCLC-C HCC.

However, our study had some limitations. First, all of our patients were from China. Most of our patients progressed by HBV infection. Second, the nomogram was built and internally validated in a single centre. Further evaluation requires external validation. Third, we extracted our radiomic features only from arterial phase-enhanced CT. Combined with portal-venous and delayed phase may provide more effective radiomic features. Furthermore, a multicenter prospective study with a larger sample may reduce our biases study and confirm the nomogram's stability and efficacy.

In conclusion, the predictive nomogram that combines radiomic features with clinical risk factors has great prospects for application in the prediction of post-SBRT OS in BCLC-C HCC patients.

## Figures and Tables

**Table 1:** Characteristics of the patient.

Characteristics	Participants (*n*)			
	Total (*n* = 137)	Training set (*n* = 95)	Validation set (*n* = 42)	*p* value
Age				0.87
<50 years	48	34	14	
≥50 years	89	59	28	
Sex				0.08
Male	120	82	38	
Female	17	13	4	
HBsAg				0.10
Negative	18	16	2	
Positive	119	79	40	
CTP class				0.47
A	86	62	24	
B	51	33	18	
Performance status				1.00
1	120	83	37	
2	17	12	5	
Largest tumor size				0.20
≤5 cm	55	42	13	
>5 cm	82	53	29	
Pre-SBRT AFP				1.00
≤20 ng/mL	31	21	10	
>20 ng/mL	106	74	32	
MVI				0.45
No	8	7	1	
Yes	129	88	41	
N stage				0.16
0	81	52	29	
1	53	43	13	
Number of tumors				0.85
≤3	75	53	22	
>3	62	42	20	
Prior/combined TACE				0.97
No	21	14	7	
Yes	116	81	35	
Prior/combined use of systemic therapy				0.59
No	12	7	5	
Yes	125	88	37	

The *p* values are derived from comparisons between the training and validation sets.

**Table 2:** Univariate and multivariable Cox regression analysis for the prognostic factor of overall survival.

Variable	Univariate analysis		Multivariable analysis	
	HR (95% CI for HR)	*p*	HR (95%CI for HR)	*p*
Rad-score	11.87 (5.19, 27.13)	<0.001	10.88 (4.43, 26.75)	<0.001
Tumor size	2.06 (1.32, 3.20)	0.001	1.63 (1.01, 2.63)	0.04
BED	0.41 (0.27, 0.63)	<0.001	0.46 (0.30, 0.71)	<0.001
NOT	1.96 (1.3, 2.9)	0.001	1.53 (0.97, 2.39)	0.06
MVI	2.05 (0.75, 5.6)	0.160		
CTP	2.36 (1.55, 3.59)	<0.001	1.70 (1.09, 2.64)	0.02
PS	1.09 (0.58, 2.04)	0.79		
N	0.73 (0.48, 1.11)	0.14		
AFP	1.24 (0.96, 1.62)	0.10		
Hepatitis	0.74 (0.44, 1.24)	0.25		
AGE	1.01 (0.99, 1.03)	0.11		
SEX	1.13 (0.61, 2.07)	0.67		

**Table 3:** *C*-index comparison between the nomogram and all models.

Methods	Training set		Validation set	
	*C*-index (95% CI)	*p* value	*C*-index (95% CI	*p* value
Nomogram	0.77 (0.72–0.82)	—	0.71 (0.61–0.81)	—
Radiomics signature	0.67 (0.59–0.74)	<0.01	0.66 (0.55–0.77)	0.10
Clinical model	0.72 (0.66–0.78)	<0.05	0.67 (0.57–0.77)	0.12

## Data Availability

The de-identified individual-level data are available from the authors upon reasonable request.

## Abbreviations

3D: 3 dimensional
AFP: Alpha-fetoprotein
AUC: Area under the curve
BCLC-C: Barcelona Clinic Liver Cancer stage C
BED: Biological effective dose
CT: Computed tomography
CTP: Child-Turcotte-Pugh
DCA: Decision curve analysis
HCC: Hepatocellular carcinoma
ICIs: Immune checkpoint inhibitors
ICC: The intergroup correlation coefficient
LASSO: Least absolute shrinkage and selection operator
MRI: Magnetic resonance imaging
MVI: Macrovascular invasion
PS: Performance status
ROC: Receiver operating characteristic curve
SBRT: Stereotactic body radiotherapy
TACE: Transarterial chemoembolization.

## Supplementary Materials

Supplementary Materials Supplementary Figure 1: Flowchart of the patient selection process. Supplementary Figure 2: The ROI segmentation of a patient.
