# Supplementary material for: Prognostic Value of a CT Radiomics-Based Nomogram for the Overall Survival of Patients with Nonmetastatic BCLC Stage C Hepatocellular Carcinoma after Stereotactic Body Radiotherapy
Source: J Oncol. 2023 Jan 3;2023:1554599. doi: 10.1155/2023/1554599 (PMC9831699; doi:10.1155/2023/1554599)
Supplement: Supplementary Materials — Supplementary Figure 1: Flowchart of the patient selection process. Supplementary Figure 2: The ROI segmentation of a patient. [file 1554599.f1.zip › Supplementary figure 1.docx]

Validation Cohort (n=42)

Training Cohort (n=95)

Whole Cohort (n=137)

Exclusion

•Examined by unassigned CT scanners

(n =18)

•Received previous Radiotherapy (n =5)

•Clinical data were incomplete (n =22)

•Difficulty in distinguishing boundary of ROIs (n=7)

BCLC-C HCC
patients from from December 2016 to September 2020 (n=189)
