# Supplementary figures and images for: Prognostic Value of a CT Radiomics-Based Nomogram for the Overall Survival of Patients with Nonmetastatic BCLC Stage C Hepatocellular Carcinoma after Stereotactic Body Radiotherapy
Source: J Oncol. 2023 Jan 3;2023:1554599. doi: 10.1155/2023/1554599 (PMC9831699; doi:10.1155/2023/1554599)

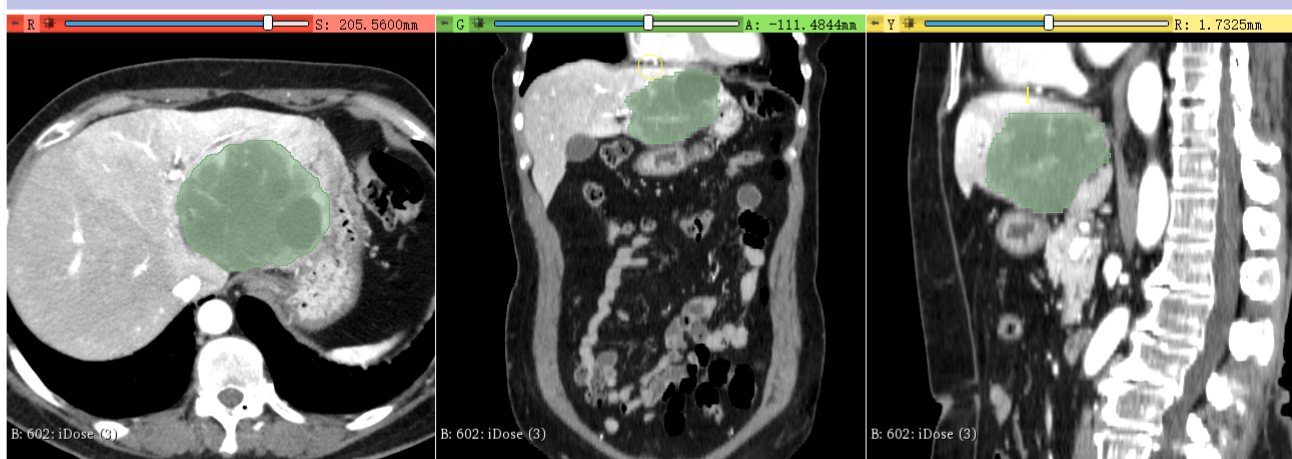

Supplement: Supplementary Materials — Supplementary Figure 1: Flowchart of the patient selection process. Supplementary Figure 2: The ROI segmentation of a patient. [file 1554599.f1.zip › Supplementary figure 2.docx]
